# Prevalence and correlates of occupational noise-induced hearing loss among workers in the steel industry

**DOI:** 10.1186/s42506-023-00135-7

**Published:** 2023-06-05

**Authors:** Noha Elshaer, Dorria Meleis, Abdelrahman Mohamed

**Affiliations:** 1 Industrial Medicine and Occupational Health, Community Medicine Department, Faculty of Medicine, Alexandria University, Alexandria, Egypt; 2 Preventive Medicine Sector, Ministry of Health and Population, Alexandria, Egypt

**Keywords:** Hearing impairment, Noise-induced hearing loss, Tinnitus

## Abstract

**Background:**

The steel industry is one of the noisiest industries, which can predispose workers to hearing loss. In Egypt, the demand for steel is increasing due to the construction of new infrastructures as bridges, flyover roads, buildings, and towers; however, little is known about the prevalence of occupational noise-induced hearing loss (NIHL) among steel workers. Understanding the distribution of the affected workers is crucial for planning prevention strategies. This study aimed to estimate the prevalence of occupational NIHL among Egyptian steel workers and identify its correlates.

**Methods:**

This study was conducted at two steel factories in Egypt in November 2021. It involved an initial retrospective review of the factory medical records of the latest periodic medical examination conducted on workers from July to September in the year 2021 representing workers’ health status in that year. Then, a case–control approach analysis was carried out. Eligible workers (*n* = 606) were enrolled and divided into two groups: noise-exposed workers (*n* = 396) and unexposed workers (*n* = 210). Univariate and multivariate regression analyses were performed.

**Results:**

Occupational exposure to hazardous A-weighted equivalent noise level (> 85 dB) was associated with higher hearing thresholds at all frequencies (highest at 4 kHz followed by 6 kHz), particularly in younger workers below the age of 40 years. Nearly 71% of noise-exposed workers had hearing impairment, and 47% had NIHL compared with unexposed workers (45.7% and 11.9%, respectively). The probability of NIHL in noise-exposed workers was 6.55 times higher than that in unexposed workers (OR = 6.55, 95%CI = 4.13, 10.40; *p* < 0.001). In noise-exposed workers, age and tinnitus were independent predictors of hearing thresholds, while tinnitus was found to be an independent predictor of NIHL after adjusting for age and job duration (OR = 2.06, 95%CI = 1.01, 4.20; *p* = 0.045).

**Conclusion:**

Almost half of noise-exposed workers had NIHL. Tinnitus was found to be an independent predictor of NIHL. Decreasing noise exposure levels in steel plants is recommended to reduce hearing loss. Future research is required to study the effect of tinnitus on audiometry measurements among workers with NIHL.

**Supplementary Information:**

The online version contains supplementary material available at 10.1186/s42506-023-00135-7.

## Introduction

Globally, the demand for steel is increasing due to the construction of new infrastructures such as bridges, flyover exchange roads, buildings, towers, and railways. This sector has provided employment for millions of people [1]. In the iron and steel industry, steelmaking involves iron refinement, casting, and rolling mills which involves passing the metal stock between one or more pairs of rollers to reduce and uniformize thickness and impart a desired attribute [2]. While the steel industry is considered one of the most important industries in each country, steel plants are also considered some of the noisiest plants in the manufacturing sector. Among the major sources of noise in steel plants: compressors, blowers, induced draught fans, conveyors, pneumatic tools and equipment, and operations such as grinding, arcing, crushing, and rolling [3].

Excessive noise exposure at work can result in a sensory neural injury to the inner ear that leads to a partial or complete bilateral hearing loss. Occupational noise-induced hearing loss (NIHL) develops slowly over several years of exposure at work to continuous or intermittent noise exceeding the recommended exposure limit (action level) of 85 dB(A) during an 8-h shift, or impact noise exceeding 120 dB(A) during an 8-h working shift [4–7]. It affects hearing thresholds at higher frequencies; however, with further exposure, thresholds at lower frequencies can also be affected [5, 8, 9].

According to a review by Chen et al. (2020) which included 105 studies, the burden associated with occupational noise varies widely across countries and occupations globally, ranging from 11.2 to 58% and is one of the most reported occupational diseases, particularly in the less developed regions in the world [10]. Males are more affected by exposure to occupational noise than females [11]. Worldwide, it has been reported that occupational noise exposure is responsible for 16% of cases of disabling hearing loss in adults. NIHL impairs communication and can lead to social isolation resulting in a lower quality of life. In addition, it has a financial burden on the employees, employers, and society [10, 12, 13].

In less developed countries, such as Egypt, little is known about the prevalence of occupational NIHL in the steel industry [14]. Since occupational NIHL is a complex and preventable disease [10], understanding the distribution of affected workers is crucial for policymakers and stakeholders who plan for preventive services. This study was conducted to estimate the prevalence of occupational NIHL among workers engaged in the steel industry and identify factors associated with it. In addition, the study compared hearing thresholds between workers exposed to hazardous occupational noise and unexposed workers. Furthermore, this study calculated the percentage of hearing disability among noise-exposed workers.

## Methods

### Study design and setting

A retrospective design was adopted for this study. Medical records of the latest periodic medical examination of workers at two steel factories in Egypt were reviewed in November 2021. The medical examination was conducted from July to September of the same year and represents the health status of the workers in that year.

### Participants

The medical records of the selected factories included data of 709 workers, all of whom were men except for four workers. The inclusion criteria set for enrollment in the current study were male workers who had a job duration equal to or more than 5 years. Women workers and those with any condition considered as confounders to NIHL were excluded. Accordingly, 103 workers were excluded because of job duration less than 5 years (*n* = 88), previous ear infections (*n* = 5), history of exposure to noise during military service (*n* = 3), family history of hearing impairment (*n* = 2), and conductive hearing loss (air–bone gap > 10 dB) (*n* = 1). Additionally, women workers (*n* = 4) were excluded.

The 606 eligible workers were divided into two groups based on noise exposure: (i) workers exposed to hazardous occupational noise, necessitating actions (action level), where the workplace area A-weighted equivalent noise level was equal to or more than 85 dB and (ii) unexposed workers employed at non-manufacturing departments at the same factories who were not exposed to hazardous occupational noise. Exposed workers were further categorized according to audiometry results into workers with NIHL or free from NIHL.

### Power analysis

A power analysis was conducted (using the Open-Epi online calculator Version 3.3a, OpenEpi, Atlanta, GA, USA). It showed that enrolling 396 noise-exposed workers and 210 unexposed workers is capable of detecting the least difference in prevalence of NIHL of 17% between both groups [14], with a prevalence ratio of 1.5 at a power of 98.2% and confidence level of 0.95 (*α* = 0.05).

### Data collection

A transfer sheet was designed to retrieve relevant data from the records. The sheet included the following:

#### Sociodemographic, medical, and occupational characteristics

Workers’ medical records were reviewed for sociodemographic data (such as age, residence, highest educational attainment, marital status, and smoking status); occupational data (including job duration, job nature, work schedule, department, and occupation); and medical condition (such as ear related medical conditions, and tinnitus).

#### Results of pure tone audiometry testing

In the periodic medical examination at the selected factories, a pure-tone audiometer was used to assess hearing acuity [15] for both ears at eight octave intervals: using ascending pure tones at frequencies of 0.5, 1, 2, 3, 4, and 6 kHz, and a range of intensity of − 10 to 120 dB. The mean threshold values at 0.5, 1, and 2 kHz were used to determine low-frequency hearing status, while the mean threshold values at 3, 4, and 6 kHz were used to determine high-frequency hearing status. On a certain test frequency, normal hearing was defined as binaural hearing level ≤ 25 dB [5].

In this study, according to the World Health Organization (WHO) noise exposure guidelines, hearing impairment was defined as a hearing threshold > 25 dB at any examined frequency (either monaural or binaural hearing impairment) [4, 5]. The audiometric ISO values (averages of values at 0.5, 1, 2, and 4 kHz) were used to categorize hearing impairment as follows: slight impairment (audiometric ISO value 26–40); moderate (ISO value 41–60 dB), severe (ISO value 61–80 dB), and profound (ISO value 81 dB or greater). NIHL was defined as a notch shown at 4 kHz (around 3 to 6 kHz) and threshold values at high-frequency worse than threshold values at low frequency [5].

#### Calculated percentage of hearing disability

The percentage of hearing disability was calculated for each worker according to the Egyptian occupational health standards (OHS) formula [16] as follows: first, the average hearing threshold level at 0.5, 1, and 2 kHz was calculated for each ear. Then, the percent impairment for each ear was calculated by multiplying the amount by which the above average hearing threshold level exceeds 25 dB by 100/65 up to a maximum of 100%, which is reached at 90 dB. Binaural assessment was calculated by multiplying the smaller percentage (better ear) by 5, adding this figure to the larger percentage (poor ear), and dividing the total by 6 [16]. For each worker, the calculated percentage of hearing disability was compared with that calculated using the American Academy of Otolaryngology and American Council of Otolaryngology (AAO-ACO) formula [17].

#### Workplace area A-weighted equivalent noise level measurement

Factory records were reviewed to obtain measurements of workplace area A-weighted equivalent noise level. At both factories, measurement was done using a sound pressure level noise meter (3 M™ Sound Detector SD-200), manufactured according to the International Standard Classifications [IEC 61,672–1 (2002), IEC 61,010–1 (2010), ANSI S1.4 1983 (R2006), ANSI S1.43 (R2007), CE]. The A-weighted network was selected, and the sound pressure level meter was calibrated before use. Multiple readings were recorded during the shift, then the average noise level was calculated (in dB) for each workplace area. At workplace areas with an A-weighted equivalent noise level ≥ 85 dB (such as the compressors room; turning workshop, welding workshop, tying machine, and mechanical maintenance workshop), workers were considered as noise-exposed workers. Whereas at workplace areas with an A-weighted equivalent noise level < 85 dB (such as the billet charging area, reheating furnace control room, repair workshop, billet storage yard, and quality control lab), workers were considered unexposed.

### Statistical analysis

The SPSS v.22 (IBM Corp. Released 2011. IBM SPSS Statistics for Mac, Armonk, NY, USA) was used for data analysis. Descriptive statistics were used to present qualitative data (frequencies and percentages) and quantitative data (mean and standard deviation). Data analysis involved an initial comparison between noise-exposed workers and unexposed workers to identify the frequency of occupational NIHL. Then, among noise-exposed workers, a case–control approach analysis was carried out to determine factors associated with NIHL.

Among all workers in the study (*n* = 606), hearing thresholds (dB) at specified tested audiometry frequencies (Hz), and ISO values were presented using mean and standard deviation for both noise-exposed and unexposed workers, stratified by age into four groups (< 30, 30 to < 40, 40 to < 50, and ≥ 50 years). The prevalence of hearing impairment and NIHL were calculated. A case–control approach analysis using univariate logistic regression was conducted to compute odds ratio (OR) and associated 95%CI to quantify the probability of hearing impairment or NIHL (dependent variable) associated with noise exposure (independent variable).

Among noise-exposed workers (*n* = 396), the mean hearing thresholds was calculated among the four age groups and three job duration groups (< 10, 10 to < 20, and ≥ 20 years). Multiple linear regression analysis was used to determine predictors of hearing threshold at the tested frequencies. In addition, univariate logistic regression was conducted to compute the odds of NIHL (dependent variable) associated with each sociodemographic, occupational, and medical factor (independent variables). Subsequently, multivariate logistic regression was conducted to model NIHL as a function of the significant factors identified in the univariate analysis, namely age, job duration, and tinnitus, to study their independent effect. The adequacy of the model in data fitting was determined using Nagelkerke’s *R*^2^ and Hosmer and Lemeshow goodness-of-fit test. All statistical analyses were judged at a level of significance of 5% (*α* = 0.05).

As for workers with hearing disability (> 0%), a comparison was made between the mean percentage of hearing disability calculated using the Egyptian formula and AAO-ACO formula.

## Results

### Sociodemographic, medical, and occupational characteristics (*n* = 606)

Among the enrolled workers, 64.7% were below the age of 40, 75% lived in urban areas, 60% had attained a high school education, 87% were married, and 34% had never smoked. Seventy-seven percent of workers were blue-collar workers, 71.9% had a job duration of less than 10 years, and 67.7% worked in shifts. Thirty-nine percent of the studied workers worked at the production department, and 40% were production technicians and supervisors (Table 1).

**Table 1:** Sociodemographic and workplace characteristics of the studied workers at two steel factories in Egypt, 2021 (*n* = 606)

Characteristic	Frequency (no.)	Percentage (%)
Age (years)		
<30	58	9.6
30– < 40	334	55.1
40– < 50	145	23.9
≥ 50	69	11.4
Residence		
Rural	150	24.8
Urban	456	75.2
Highest educational attainment		
Never been to school	12	2.5
Basic education	48	7.9
High school	363	59.9
Higher education	180	29.7
Marital status		
Never married	61	10.1
Married	527	87.0
Divorced	18	3.0
Smoking status		
Never been smoker	206	34.0
Ex-smoker	66	10.9
Current smoker	334	55.1
Job duration (years)		
< 10	436	71.9
10– < 20	105	17.3
≥ 20	65	10.7
Job nature		
Blue collar	469	77.4
White collar	127	21.0
Pink collar	10	1.7
Work schedule		
Daytime work	196	32.3
Shiftwork	410	67.7
Department		
Production	236	38.9
Maintenance	82	13.5
Administrative affairs	65	10.7
Electricity	37	6.1
Storage	31	5.1
Quality	27	4.5
Facilities	23	3.8
Others^a^	105	17.3
Occupation		
Production technician	124	20.4
Production supervisor	119	19.6
Maintenance technician	59	9.7
Department manager	28	4.6
Store keeper	24	4.0
Electric technician	21	3.5
Engineer	20	3.3
Oven operator or technician	19	3.1
Welding technician	19	3.1
Rolling technician	18	3.0
Workshop technician	18	3.0
Administration personnel	18	3.0
Others^b^	119	19.6

^a^Including technical (2.8%), security (2.1%), industrial safety and health (2.0%), fleet (2.0%), deliverables and sales (1.8%), medical care (1.7%), balance (1.5%), purchases (1.3%), hydraulic (1.2%), and mechanics departments (1.0%)

^b^Including quality technician (2.6%), driver (2.6%), accountant (2.1%), crane operator (1.8%), service worker (1.8%), physician/nursing specialist (1.8%), quality specialist (1.5%), security personnel (1.3%), balance operator (1.3%), sales personnel (1.2%), human resources specialist (0.7%), public relationship specialist (0.5%), and lawyer (0.3%)

No significant differences were found between noise-exposed workers (*n* = 396) and unexposed workers (*n* = 210) with respect to age, job duration, smoking status, ear-related medical conditions, or tinnitus (Table 2).

**Table 2:** Characteristics of noise-exposed workers (*n* = 396) and unexposed workers (*n* = 210) at two steel factories in Egypt, 2021

Characteristics	Noise-exposed workers (*n* = 396)		Unexposed workers (*n* = 210)		*P* value
	No.	%	No.	%	
Job duration (years)					
< 10	277	69.9	159	75.7	0.240^a^
10– < 20	76	19.2	29	13.8	
≥ 20	43	10.9	22	10.5	
Smoking status					
Never been smoker	128	32.3	78	37.1	0.061^a^
Ex-smoker	37	9.3	29	13.8	
Current smoker	231	58.3	103	49.0	
Ear-related medical condition					
No	362	91.4	194	92.4	0.681^b^
Yes	34	8.6	16	7.6	
Tinnitus					
No	356	89.9	198	94.3	0.067^b^
Yes	40	10.1	12	5.7	
Age (years)					
< 40	252	63.6	140	66.7	0.458^b^
≥ 40	144	36.4	70	33.3	
Mean ± SD (Min–Max)	38.41 ± 7.24 (25–62)		38.05 ± 8.88 (24–63)		0.594^c^

*Abbreviations*: *SD* Standard deviation

^a^Monte Carlo test

^b^Chi-square test

^c^Student’s *t* test

### Hearing threshold (dB) at tested audiometry frequencies (0.5, 1, 2, 3, 4, and 6 kHz)

#### Hearing threshold among all studied workers (*n* = 606)

**Fig. 1:**
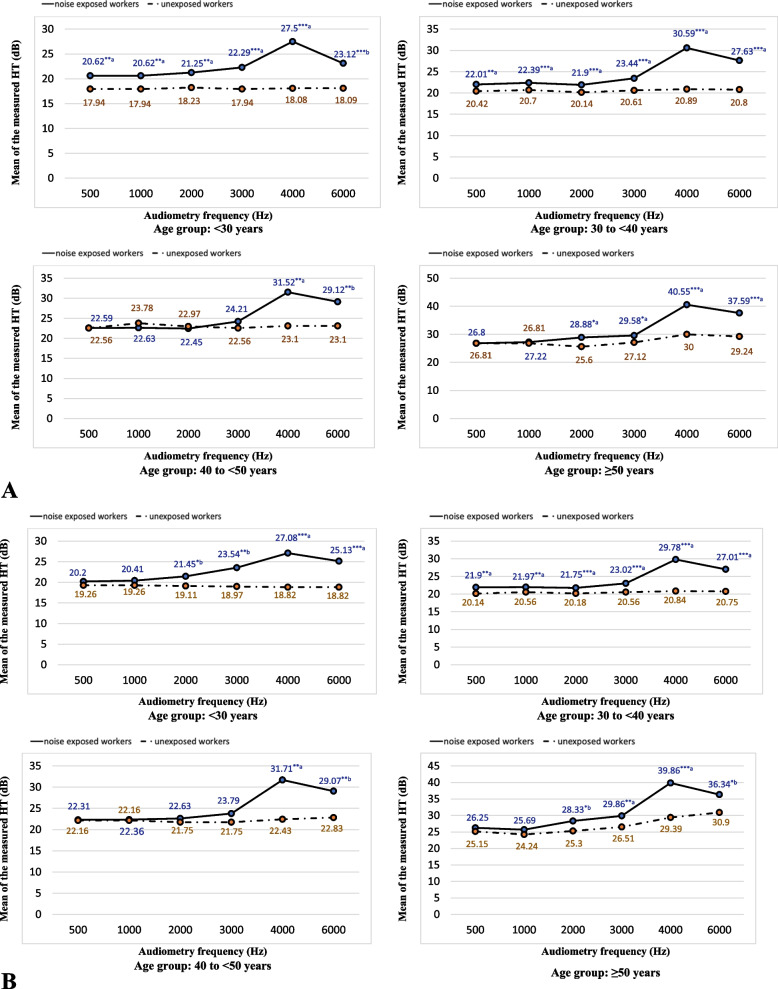
The mean of the measured hearing threshold (dB) at specified audiometry frequencies (Hz) among noise-exposed (*n* = 396) and unexposed workers (*n* = 210) stratified by the age group, at two steel factories in Egypt, 2021. Abbreviations: dB decibel, Hz Hertz. ^a^Mann-Whitney *U* test; ^b^Student’s *t* test; ^*^p < 0.05; ^**^p < 0.01; ^***^p < 0.001. **A**: right ear; **B**: left ear

Noise-exposed workers had significantly higher mean hearing threshold compared with unexposed workers at all tested frequencies (Table 3). With respect to the four age-groups, workers below the age of 40 years had a significantly higher mean hearing threshold at most of the tested frequencies, whereas workers at or above 40 years had a significantly higher mean hearing threshold principally at 4 kHz and 6 kHz compared to unexposed workers (Fig. 1). In all age groups, noise-exposed workers had significantly higher high-frequency threshold values and ISO values than unexposed workers (Figure S1).

**Table 3:** Mean hearing threshold (dB) at tested audiometry frequencies (Hz) among noise-exposed workers (*n* = 396) and unexposed workers (*n* = 210) at two steel factories in Egypt, 2021

	Number	Mean hearing threshold (dB) for tested audiometry frequency (kHz) Mean ± SD					
		0.5 kHz	1 kHz	2 kHz	3 kHz	4 kHz	6 kHz
**Right ear**							
Noise-exposed	396	22.52 ± 5.31^a**^	22.79 ± 5.09^a**^	22.65 ± 5.39^a***^	24.14 ± 6.23^a***^	31.56 ± 13.9^a***^	28.67 ± 11.5^a***^
Unexposed	210	21.40 ± 6.03	21.76 ± 6.08	21.19 ± 5.89	21.54 ± 6.36	22.26 ± 8.66	22.09 ± 8.42
**Left ear**							
Noise-exposed	396	22.31 ± 5.45^a**^	22.32 ± 5.00^a**^	22.57 ± 5.51^a***^	23.88 ± 6.35^a***^	31.06 ± 13.2^a***^	28.30 ± 10.8^a***^
Unexposed	210	21.14 ± 5.50	21.21 ± 5.45	21.09 ± 5.96	21.45 ± 6.27	22.14 ± 8.03	22.40 ± 8.38

*Abbreviations*: *SD* Standard deviation, *dB* Decibel, *Hz* Hertz

^**^p value < 0.01

^***^p value < 0.001

^a^Mann-Whitney *U* test

#### Hearing threshold among noise-exposed workers (*n* = 396)

The highest mean hearing threshold was reported at 4 kHz (31.56 ± 13.9 dB), followed by 6 kHz (28.67 ± 11.5) (Table 3). The mean high-frequency hearing threshold was higher than the mean low-frequency hearing threshold in all age groups (Figure S2) and job duration groups (Figure S3).

### Prevalence of hearing impairment and noise-induced hearing loss (NIHL)

According to the audiometry results, 71.2% of noise-exposed workers exhibited unilateral or bilateral hearing impairment, and 47% were diagnosed as having unilateral or bilateral NIHL compared with unexposed workers (45.7% and 11.9%, respectively). Univariate analysis revealed a significant association between noise exposure at work and binaural hearing impairment and NIHL. The probability of binaural hearing impairment in noise-exposed workers was 2.93 times higher than that in unexposed workers (OR = 2.93, 95%CI = 2.07, 4.16; *p* < 0.001). In addition, the probability of binaural NIHL in noise-exposed workers was 6.55 times higher than that in unexposed workers (OR = 6.55, 95%CI = 4.13, 10.40; *p* < 0.001) (Table 4).

**Table 4:** Prevalence of hearing impairment and NIHL among noise-exposed (*n* = 396) and unexposed workers (*n* = 210) at two steel factories in Egypt, 2021

	Noise-exposed workers (*n* = 396)		Unexposed^^^ workers (*n* = 210)		OR^b^ (95% CI)	*P* value^a^
	No.	%	No.	%		
**Right ear**						
Hearing impairment						
No	45	11.4	59	28.1	3.04 (1.97, 4.69)	< 0.001^***^
Yes	351	88.6	151	71.9		
Slight	347		147			
Moderate	4		4			
NIHL						
No	213	53.8	195	92.9	11.16 (6.37, 19.57)	< 0.001^***^
Yes	183	46.2	15	7.1		
**Left ear**						
Hearing impairment						
No	44	11.1	57	27.1	2.98 (1.92, 4.61)	< 0.001^***^
Yes	352	88.8	153	72.8		
Slight	345		151			
Moderate	6		2			
Severe	1		0			
NIHL						
No	216	54.5	189	90.0	7.50 (4.58, 12.27)	< 0.001^***^
Yes	180	45.5	21	10.0		
**Binaural**						
Hearing impairment						
No	114	28.8	114	54.3	2.93 (2.07, 4.16)	< 0.001^***^
Yes	282	71.2	96	45.7		
NIHL						
No	210	53.0	185	88.1	6.55 (4.13, 10.40)	< 0.001^***^
Yes	186	47	25	11.9		

Abbreviations: *NIHL* Noise-induced hearing loss, *OR* Odds ratio, *CI* Confidence interval

Hearing impairment is categorized according to audiometric ISO value (average of values of hearing thresholds at 0.5, 1, 2, and 4 kHz) into slight (20–40 dB), moderate (41–60 dB), and severe impairment (61–80 dB)

NIHL is defined as a notch shown at 4 kHz (around 3 to 6 kHz), and threshold values at high-frequency substantially worse than threshold values at low frequency

^a^Chi-square test

^b^Univariate logistic regression was conducted to compute the odds of hearing impairment/NIHL (dependent variable) associated with noise exposure (independent variable)

^^^Reference

^***^p < 0.001

### Factors associated with NIHL among noise-exposed workers (*n* = 396)

In the univariate analysis, age, job duration, and tinnitus were significantly associated with NIHL. The probability of NIHL was 7 times higher among workers who were 50 years old or older, 3.78 times higher among workers with a job duration of 20 years or more, and 2 times higher among workers who had tinnitus (Table 5). In multivariate logistic regression, tinnitus was found to be an independent predictor of NIHL after adjustment of age and job duration (OR = 2.06, 95%CI = 1.01, 4.20; *p* = 0.045) (Table 6). In multiple linear regression, age and tinnitus were predictors of hearing thresholds at most of the tested frequencies (Table S1).

**Table 5:** Univariate logistic regression of factors associated with NIHL among noise-exposed workers (*n* = 396) at two steel factories in Egypt, 2021

Factors	NIHL				OR^#^ (95% CI)	*P* value
	Yes (*n* = 186)		No (*n* = 210)			
	No.	%	No.	%		
Highest qualification attained						
Never been to school^^^	3	1.6	2	1.0	-	-
Basic education	18	9.7	18	8.6	0.66 (0.09, 4.47)	0.677
High school	131	70.4	147	70.0	0.59 (0.09, 3.61)	0.572
Higher education	34	18.3	43	20.5	0.52 (0.08, 3.33)	0.496
Smoking						
Never been smoker^^^	62	33.3	66	31.4	-	-
Current/ex-smoker	124	66.7	144	68.6	0.91 (0.60, 1.39)	0.686
Ear-related medical condition						
No^^^	168	90.3	194	92.4	-	-
Yes	18	9.7	16	7.6	1.29 (0.64, 2.62)	0.467
Tinnitus						
No^^^	161	86.6	195	92.9	-	-
Yes	25	13.4	15	7.1	2.01 (1.03, 3.95)	0.041^*^
Job nature						
Blue collar^^^	185	99.5	205	97.6	-	-
White collar	1	0.5	5	2.4	0.22 (0.02, 1.91)	0.171
Work schedule						
Daytime work^^^	29	15.6	47	22.4		-
Shiftwork	157	84.4	163	77.6	1.56 (0.93, 2.60)	0.088
Using hearing PPE at work						
No^^^	93	50.0	126	60.0	-	-
Yes, regular	15	8.1	11	5.2	1.84 (0.81, 4.20)	0.144
Yes, irregular	78	41.9	73	34.8	1.44 (0.95, 2.19)	0.082
Job duration (years)						
< 10^^^	121	65.1	156	74.3	-	-
10– < 20	33	17.7	43	20.5	0.98 (0.59, 1.65)	0.968
≥ 20	32	17.2	11	5.2	3.78 (1.81, 7.74)	< 0.001^***^
Mean ± SD (Min–Max)	11.9 ± 8.4 (5–35)		9 ± 4.1 (5–27)			0.027^*a^
Age (years)						
< 30^^^	10	5.4	14	6.7	-	
30– < 40	92	49.5	136	64.8	0.94 (0.40, 2.22)	0.901
40– < 50	54	29.0	54	25.7	1.40 (0.57, 3.42)	0.461
≥ 50	30	16.1	6	2.9	7.0 (2.12, 23.11)	0.001^**^
Mean ± SD (Min–Max)	39.9 ± 8.5 (26–62)		37.1 ± 5.5 (25–55)			< 0.001^***b^

NIHL is defined as a notch shown at 4 kHz (around 3 to 6 kHz), and threshold values at high-frequency substantially worse than threshold values at low frequency

*Abbreviations*: *NIHL* Noise-induced hearing loss, *PPE* Personal protective equipment, *SD* Standard deviation, *OR* Odds ratio, *CI* Confidence interval

^#^Univariate logistic regression was conducted to compute the odds of NIHL (dependent variable) associated with each variable in the above table (independent variables)

^^^Reference: ^a^Mann-Whitney *U* test; ^b^Student’s *t* test

^*^p ≤ 0.05

^**^p < 0.01

^***^p < 0.001

**Table 6:** Multivariate logistic regression of independent predictors of NIHL among noise-exposed workers (*n * = 396) at two steel factories in Egypt, 2021

Variables	Coefficient	Adjusted OR^a^	95% CI	*P* value
Age (years)	0.028	1.028	(0.98, 1.07)	0.199
Job duration (year)	0.049	1.050	(1.00, 1.10)	0.052
Tinnitus	0.726	2.068	(1.01, 4.20)	0.045^*^

Model *X*^2^ = 24.51; *p* < 0.001; Cox & Snell *R*^2^ = 0.06; Nagelkerke’s *R*^2^ = 0.08; Hosmer & Lemeshow *X*^2^ = 13; *p* = 0.11

NIHL is defined as a notch shown at 4 kHz (around 3 to 6 kHz) and threshold values at high-frequency substantially worse than threshold values at low frequency

*Abbreviations*: *NIHL* Noise-induced hearing loss, *OR* Odds ratio, *CI* Confidence interval

^a^OR adjusted for all variables in the above table (age and job duration as continuous variables and tinnitus as a dichotomous variable)

^*^p ≤ 0.05

### Percentage of hearing disability among noise-exposed workers (*n* = 396)

**Fig. 2:**
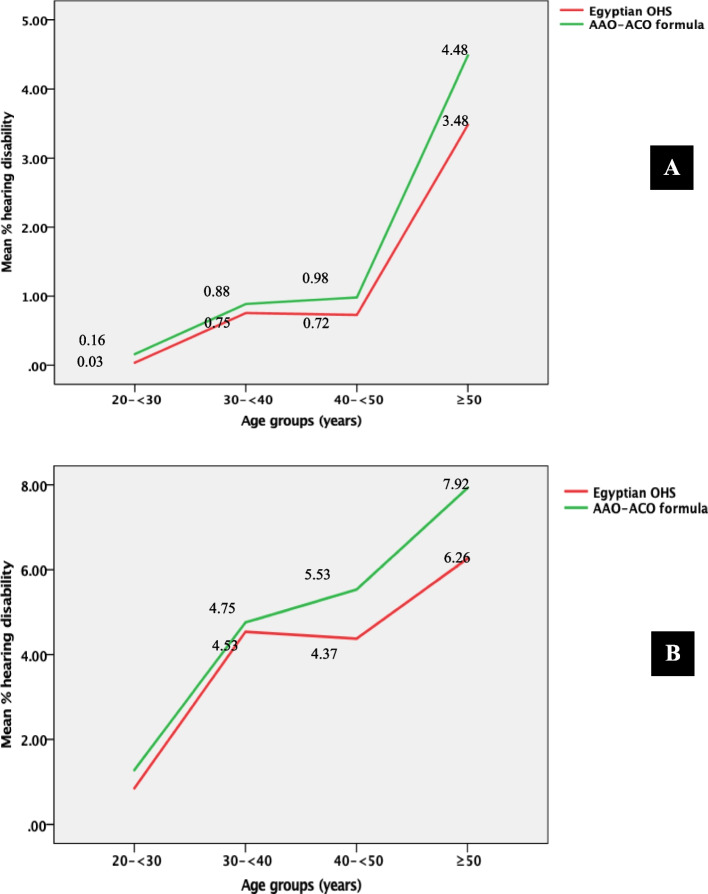
Mean percentage of hearing disability calculated according to the Egyptian OHS and AAO-ACO formulae among noise-exposed workers stratified by age, at two steel factories in Egypt, 2021. **A** Among noise-exposed workers (*n* = 396). **B** Among noise-exposed workers with % hearing disability > 0% according to the Egyptian OHS formula (*n* = 77). Abbreviations: OHS, occupational health standards; AAO-ACO, American Academy of Otolaryngology and American Council of Otolaryngology

The number of workers with a percentage of hearing disability (> 0%) was 77 (19.4%) according to the Egyptian formula and 98 (24.7%) according to the AAO-ACO formula. Among them (*n* = 77), the mean percentage of hearing disability using the Egyptian formula was similar to that calculated using the AAO-ACO formula (4.8%; 95%CI = 3.4, 6.3; and 4.8%; 95%CL = 3.5, 6.0, respectively). However, when workers were stratified by age, the mean percentage of hearing disability calculated using the AAO-ACO formula was higher than that calculated using the Egyptian OHS formula particularly among workers older than 40 years old (Fig. 2).

## Discussion

Forty-seven percent of noise-exposed steel workers in this study suffered from NIHL associated with occupational exposure. Though this figure is higher than that reported in developed countries [18], it is similar to the rates reported in Nepal (46%) [19] and Tanzania (48%) [14] and lower than the rate of 57% reported in Nigeria [20] and the rate reported in India in which it surpassed 90% [21]. The systematic review of 187 studies conducted by Lie et al. in 2016 [18] concluded that the incidence of occupational NIHL is highest in developing countries and considerably lower in industrialized countries due to the reduction of industrial noise exposure levels and the improvement of protective measures in western countries [10, 18, 22]. The use of hearing PPE by workers in the current study was not found to have protective effects. Since the results regarding the effect of using hearing protection are conflicting [18], the emphasis should be on the reduction of workplace noise exposure levels in order to reduce hearing loss among workers.

In Egypt, studies conducted among workers exposed to hazardous occupational noise showed variable results across different industries. For example, the prevalence of NIHL was 73.8% in textile workers [23], 53.5% in disc jockey workers [24], 39% in carpenters [25], and 15.3% in dry food factory workers [26]. As for Egyptian steel workers, the prevalence of hearing impairment in the current study (71.2%) was higher than that reported in an earlier study conducted in 2009 (39.6%) [27].

Tinnitus is one of the most common consequences of NIHL [28, 29] and was found to be an independent predictor of hearing thresholds and NIHL among steel workers in the current study. Tinnitus has a significant impact on quality of life and is more directly responsible for mental stress than hearing loss itself [28, 29]. In the Kang et al. study (2021), among patients with occupational NIHL, the degree of hearing loss was shown to be associated with louder tinnitus noises [30]. However, loudness of tinnitus was not reported in the current study.

Consistent with previous studies [10, 12], age was an independent predictor of hearing threshold in this study. Moreover, for workers below the age of 40 years, the mean hearing threshold at most of the tested frequencies was significantly higher in the noise-exposed group compared to the unexposed group. Similarly, as reported in a systematic review by Lie et al. [18], hearing loss among workers appeared to be the greatest during the first years of noise exposure; underscoring the heavy impact of occupational noise on the burden of hearing loss at younger ages. Younger workers who suffer from hearing loss spend more years with hearing disability, which affects the calculation of disability-adjusted life years [10], increases the risk of work injuries [31], leads to communication problems, social stress, diminished confidence, and results in bad interpersonal relationship [32, 33].

In the present study, the observed difference in the mean percentage of hearing disability calculated using the Egyptian formula and AAO-ACO formula among workers above 40 years old could be attributed to the possible effect of aging on the hearing threshold at 3 kHz (unlike the Egyptian formula [16], the AAO-ACO formula includes the hearing threshold value at 3 kHz in its calculation [17]). It is recommended that this observation be communicated to occupational health professionals in Egypt who calculate the percentage of hearing disability to determine the appropriate compensation.

### Limitations of the study

The potential variation in the effect of occupational noise exposure due to gender could not be examined in the present study due to the fact that most workers engaged in the steel industry are males. In addition, the lack of reporting on the loudness of tinnitus precluded studying its relationship with the degree of hearing loss among workers with NIHL.

## Conclusions

Occupational exposure to hazardous noise in the steel industry was found to be associated with higher hearing thresholds at all frequencies, with the highest at 4 kHz followed by 6 kHz, particularly in younger workers below the age of 40 years. Among noise-exposed workers, high prevalence of hearing impairment (71.2%) and NIHL (47%) were reported, and tinnitus was found to be an independent predictor of NIHL. The study recommends decreasing noise exposure levels in steel plants to reduce hearing loss. In addition, future research is needed to evaluate the effect of tinnitus on auditory measurements in workers with NIHL.

## Supplementary Information

**Additional file 1: Figure S1**: Mean of the mean hearing threshold at low and high audiometry frequencies, and ISO value among noise-exposed and unexposed workers stratified by age group, at two steel factories in Egypt, 2021 Abbreviations: HT: hearing threshold; dB: Decibel; Hz: Hertz. Low frequencies: average of values of HT at 0.5, 1, and 2 kHz; High frequencies: average of values of HT at 3, 4, and 6 kHz; ISO value: average of values of HT at 0.5, 1, 2, and 4kHz. ^a^Mann-Whitney U test; ^b^Student’s t test; ; *P <0.05; **P<0.01; ***P <0.001. A: right ear; B: left ear. **Figure S2.** Mean of the measured hearing thresholds at specified audiometry frequencies among noise exposed workers stratified by age, at two steel factories in Egypt, 2021 Low frequencies: average of hearing thresholds at 0.5, 1, and 2 kHz High frequencies: average of hearing thresholds at 3, 4, and 6 kHz ISO value: average of hearing thresholds at 0.5, 1, 2, and 4 kHz Abbreviations: HT: hearing threshold; dB: Decibel; Hz: Hertz A. right ear; B. left ear. **Figure S3.** Mean of the measured hearing thresholds at specified audiometry frequencies among noise-exposed workers stratified by job duration, at two steel factories in Egypt, 2021. Low frequencies: average of hearing thresholds at 0.5, 1, and 2 kHz; High frequencies: average of hearing thresholds at 3, 4, and 6 kHz; ISO value: average of hearing thresholds at 0.5, 1, 2, and 4 kHz. Abbreviations: HT: hearing threshold; dB: Decibel; Hz: Hertz A. right ear; B. left ear. **Table S1.** Multiple linear regression of predictors of hearing threshold at tested frequencies among noise-exposed workers at two steel factories in Egypt, 2021.

## Data Availability

Data are available from the corresponding author on reasonable request. Confidentiality and security of data and materials were ensured through all stages of the study.

## Abbreviations

AAO-ACO: American Academy of Otolaryngology and American Council of Otolaryngology
NIHL: Noise-induced hearing loss
OHS: Occupational Health Standards
