# Supplementary material for: Prevalence and correlates of occupational noise-induced hearing loss among workers in the steel industry
Source: J Egypt Public Health Assoc. 2023 Jun 5;98:11. doi: 10.1186/s42506-023-00135-7 (PMC10239744; doi:10.1186/s42506-023-00135-7)
Supplement: Supplementary file 1 — Additional file 1: Figure S1: Mean of the mean hearing threshold at low and high audiometry frequencies, and ISO value among noise-exposed and unexposed workers stratified by age group, at two steel factories in Egypt, 2021 Abbreviations: HT: hearing threshold; dB: Decibel; Hz: Hertz. Low frequencies: average of values of HT at 0.5, 1, and 2 kHz; High frequencies: average of values of HT at 3, 4, and 6 kHz; ISO value: average of values of HT at 0.5, 1, 2, and 4kHz. aMann-Whitney U test; bStudent’s t test; ; *P <0.05; **P<0.01; ***P <0.001. A: right ear; B: left ear. Figure S2. Mean of the measured hearing thresholds at specified audiometry frequencies among noise exposed workers stratified by age, at two steel factories in Egypt, 2021 Low frequencies: average of hearing thresholds at 0.5, 1, and 2 kHz High frequencies: average of hearing thresholds at 3, 4, and 6 kHz ISO value: average of hearing thresholds at 0.5, 1, 2, and 4 kHz Abbreviations: HT: hearing threshold; dB: Decibel; Hz: Hertz A. right ear; B. left ear. Figure S3. Mean of the measured hearing thresholds at specified audiometry frequencies among noise-exposed workers stratified by job duration, at two steel factories in Egypt, 2021. Low frequencies: average of hearing thresholds at 0.5, 1, and 2 kHz; High frequencies: average of hearing thresholds at 3, 4, and 6 kHz; ISO value: average of hearing thresholds at 0.5, 1, 2, and 4 kHz. Abbreviations: HT: hearing threshold; dB: Decibel; Hz: Hertz A. right ear; B. left ear. Table S1. Multiple linear regression of predictors of hearing threshold at tested frequencies among noise-exposed workers at two steel factories in Egypt, 2021. [file 42506_2023_135_MOESM1_ESM.docx]

|  | 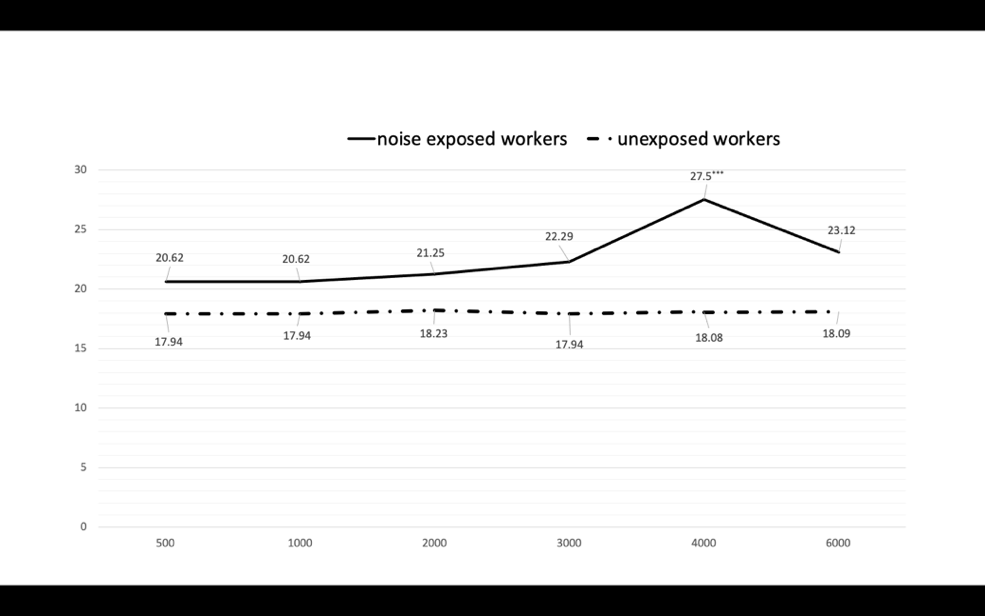 |  | 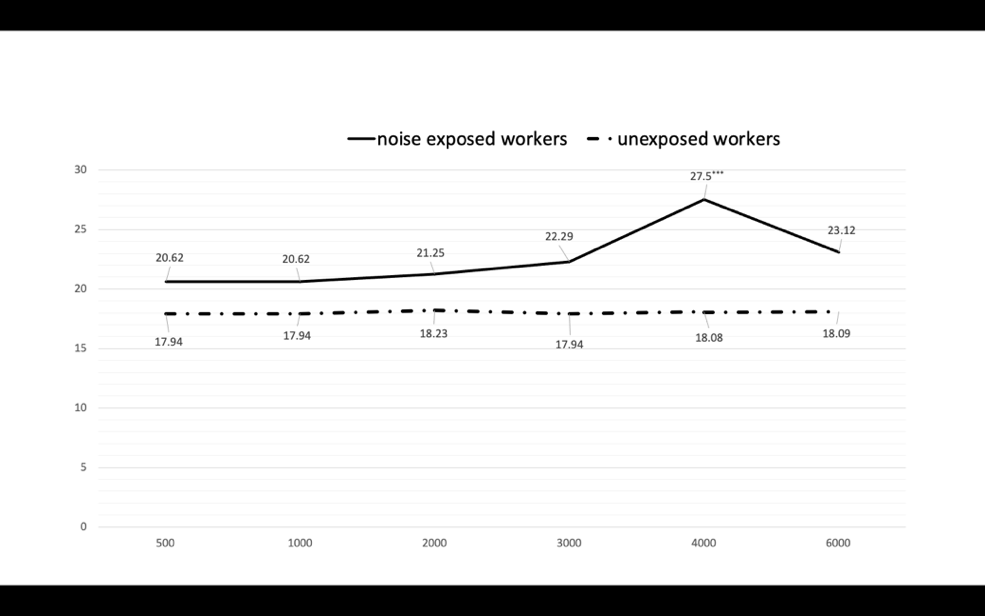 |
| --- | --- | --- | --- |
| **Mean of the mean HT (dB)** |  | **Mean of the mean HT (dB)** |  |
|  | **Audiometry frequency (Hz)** |  | **Audiometry frequency (Hz)** |
|  | **Age group: <30 years** |  | **Age group: 30 to <40 years** |
|  |  |  |  |
|  | 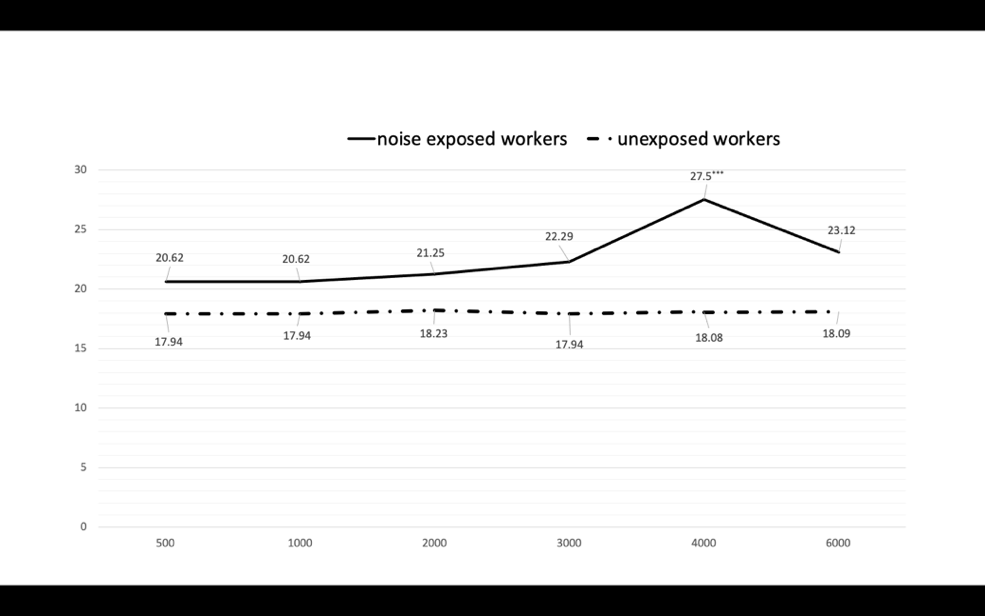 |  | 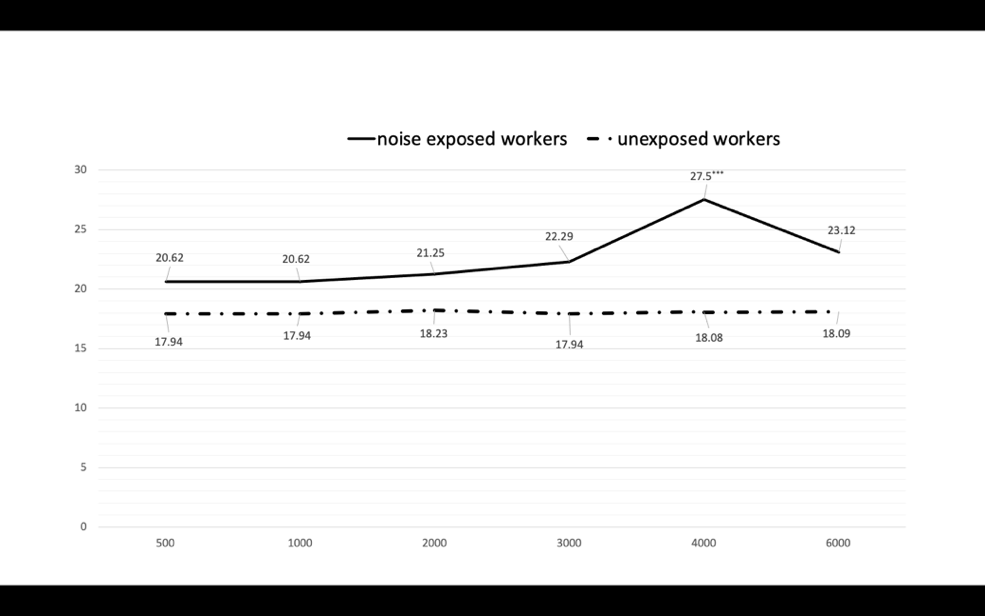 |
| **Mean of the mean HT (dB)** |  | **Mean of the mean HT (dB)** |  |
|  | **Audiometry frequency (Hz)** |  | **Audiometry frequency (Hz)** |
|  | **Age group: 40 to <50 years** |  | **Age group: ≥50 years** |
| **Figure S1.A. (Right ear): Mean of the mean hearing threshold (dB) at low and high audiometry frequencies (Hz), and ISO value among noise-exposed (n=396) and unexposed workers (n=210) stratified by age group, at two steel factories in Egypt, 2021**  Low frequencies: average of values of HT at 0.5, 1, and 2 kHz; High frequencies: average of values of HT at 3, 4, and 6 kHz; ISO value: average of values of HT at 0.5, 1, 2, and 4kHz. *Abbreviations: HT* hearing threshold; *dB* Decibel; *Hz* Hertz. ^a^ Mann Whitney U test; ^b^ Student’s t test; ^*^ p <0.05; ^**^ p<0.01; ^***^ p<0.001 | | | |

|  | 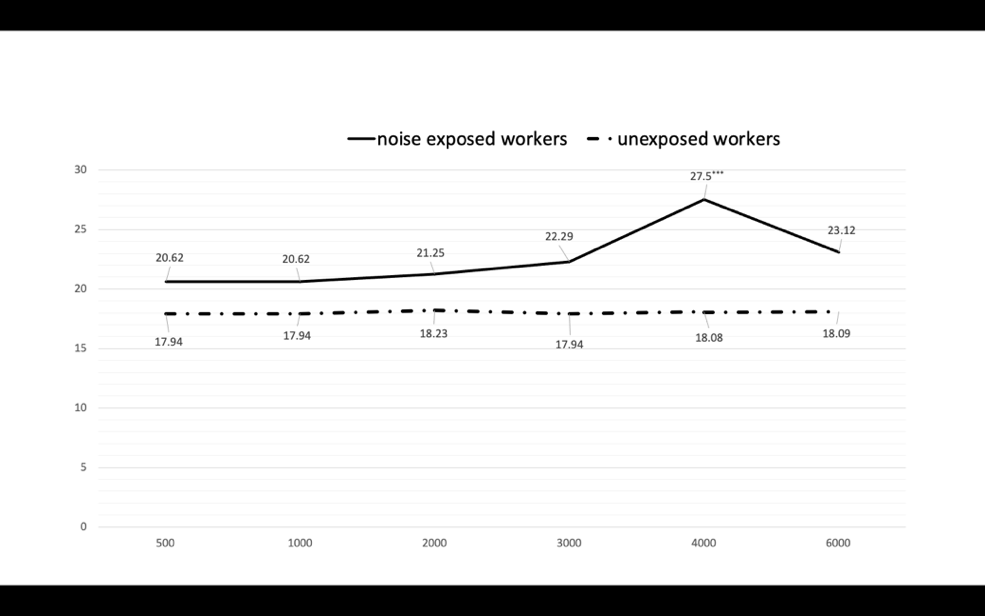 |  | 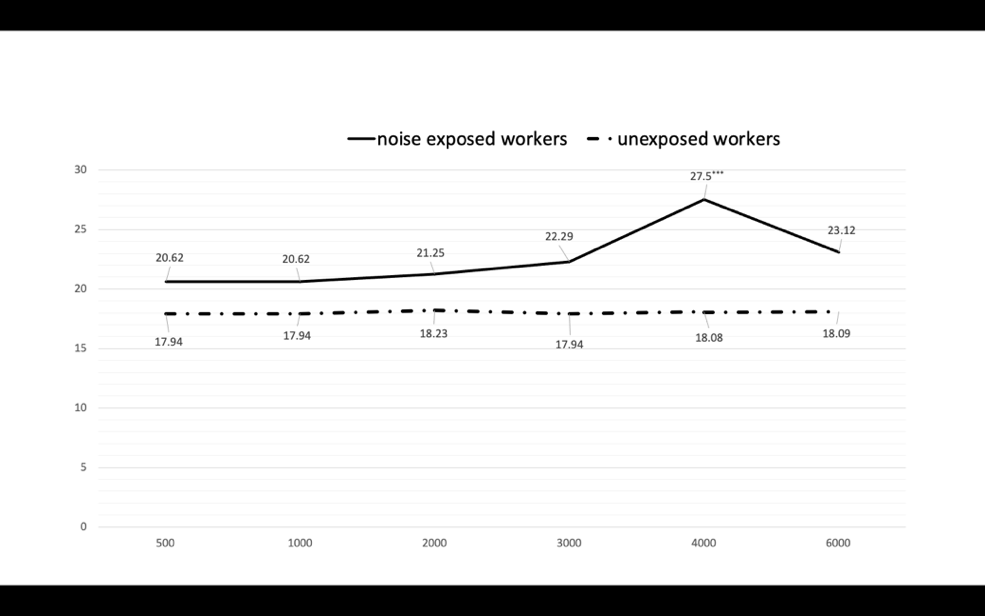 |
| --- | --- | --- | --- |
| **Mean of the measured HT (dB)** |  | **Mean of the mean HT (dB)** |  |
|  | **Audiometry frequency (Hz)** |  | **Audiometry frequency (Hz)** |
|  | **Age group: <30 years** |  | **Age group: 30 to <40 years** |
|  |  |  |  |
|  | 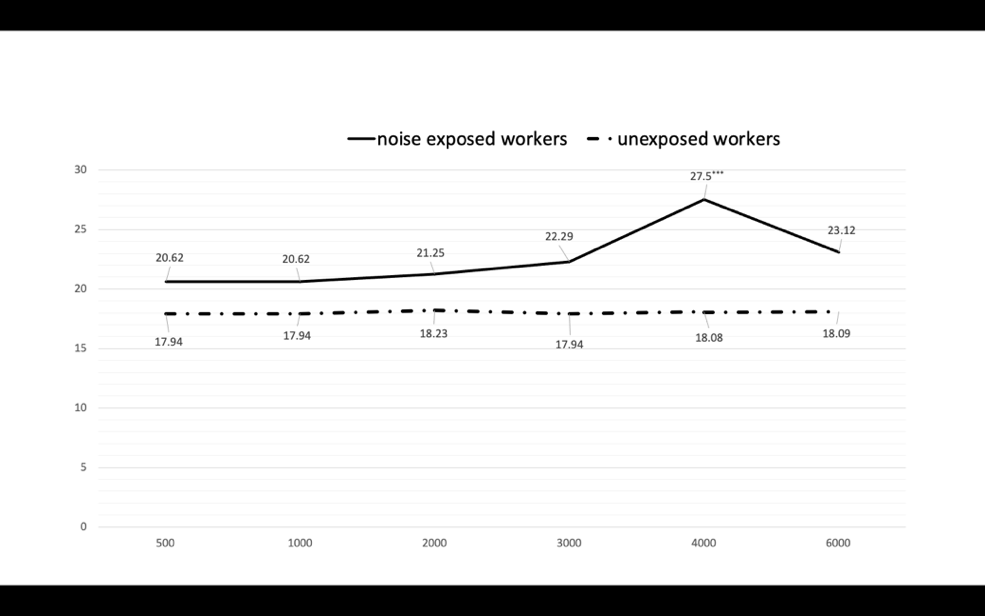 |  | 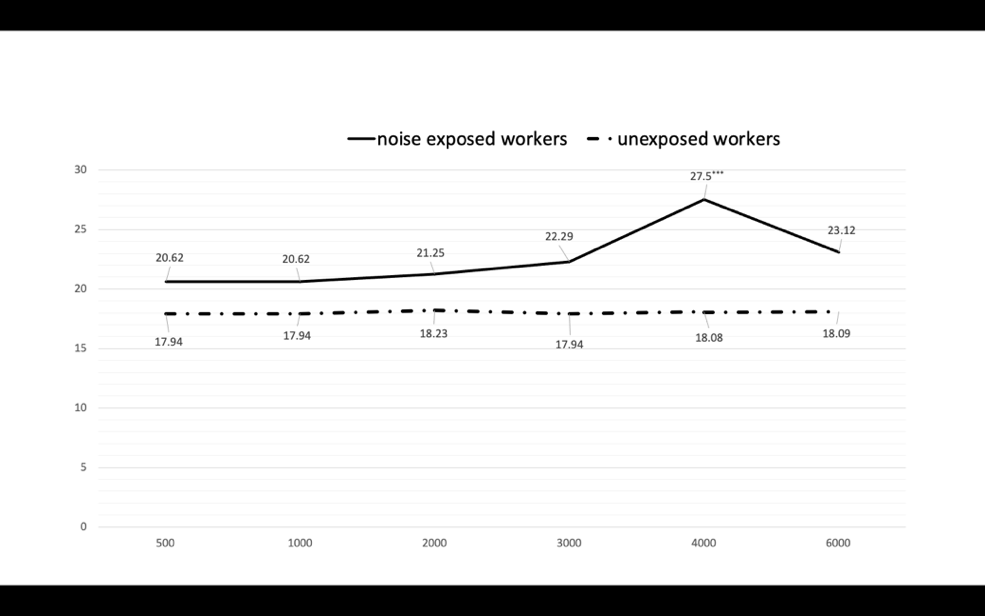 |
| **Mean of the mean HT (dB)** |  | **Mean of the mean HT (dB)** |  |
|  | **Audiometry frequency (Hz)** |  | **Audiometry frequency (Hz)** |
|  | **Age group: 40 to <50 years** |  | **Age group: ≥50 years** |
| **Figure S1.B. (Left ear): Mean of the mean hearing threshold (dB) at low and high audiometry frequencies (Hz), and ISO value among noise-exposed (n=396) and unexposed workers (n=210) stratified by age group, at two steel factories in Egypt, 2021**  Low frequencies: average of values of HT at 0.5, 1, and 2 kHz; High frequencies: average of values of HT at 3, 4, and 6 kHz; ISO value: average of values of HT at 0.5, 1, 2, and 4kHz. *Abbreviations: HT* hearing threshold; *dB* Decibel; *Hz* Hertz. ^a^ Mann Whitney U test; ^b^ Student’s t test; ^*^ p <0.05; ^**^ p<0.01; ^***^ p<0.001 | | | |

| **Mean of the measured HT** (dB) | 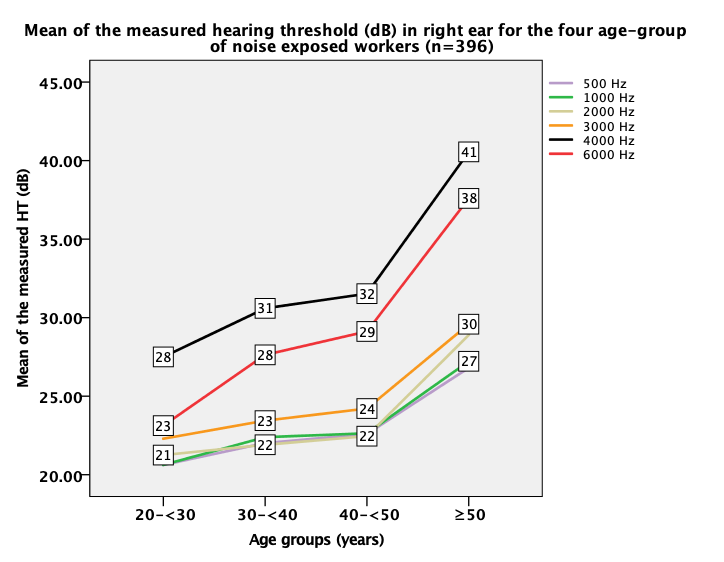 | | **Mean of the measured HT** (dB) | 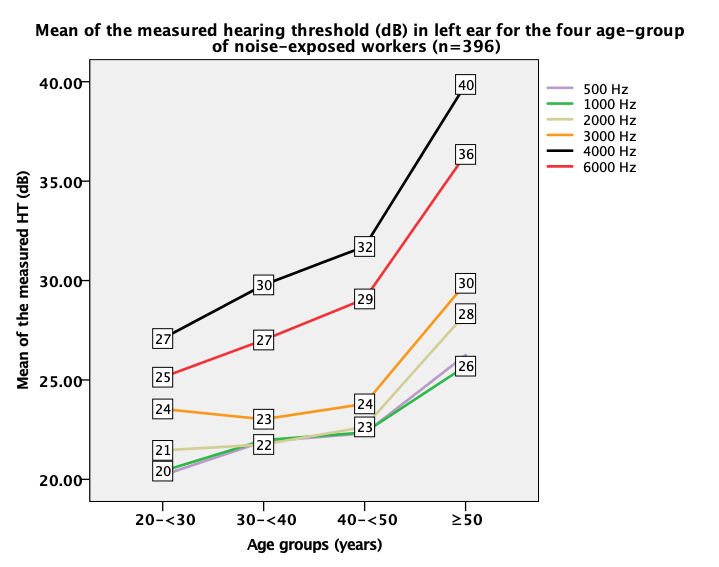 | | |
| --- | --- | --- | --- | --- | --- | --- |
|  | **A** | **Age groups** (years) |  | **B** | **Age groups** (years) | |
|  |  |  |  |  |  | |
| **Mean of the measured HT** (dB) | 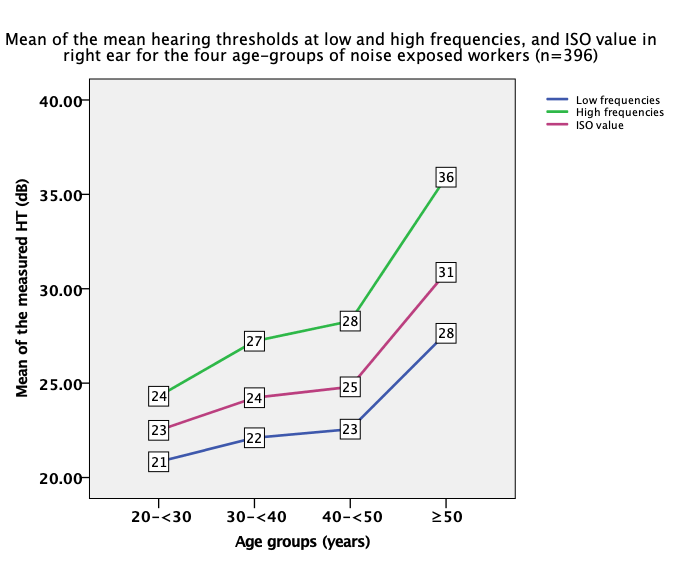 | | **Mean of the measured HT** (dB) | 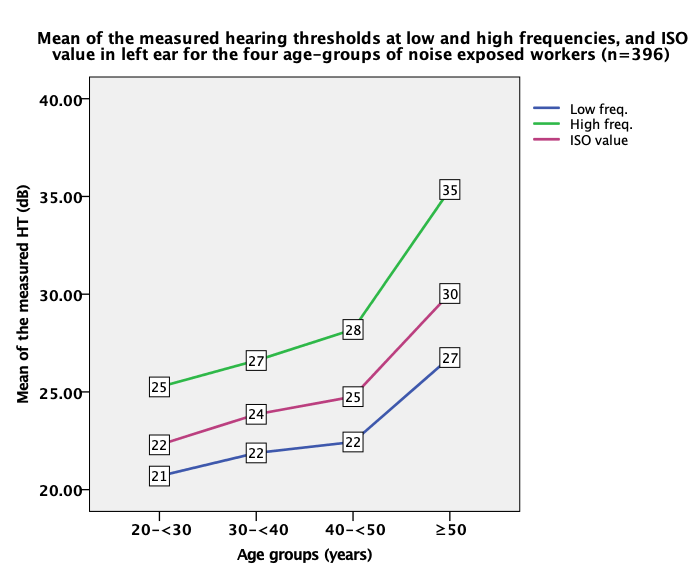 | | |
|  | **A** | **Age groups** (years) |  | **B** | **Age groups** (years) | |
|  |  | |  |  | | |
| **Figure S2: Mean of the measured hearing thresholds (dB) at specified audiometry frequencies (Hz) among noise-exposed workers (n=396) stratified by age, at two steel factories in Egypt, 2021**  Low frequencies: average of hearing thresholds at 0.5, 1, and 2 kHz  High frequencies: average of hearing thresholds at 3, 4, and 6 kHz  ISO value: average of hearing thresholds at 0.5, 1, 2, and 4 kHz  *Abbreviations*: *HT* hearing threshold; *dB* Decibel; *Hz* Hertz  A: right ear; B: left ear | | | | | |  |

| **Mean of the measured HT** (dB) | 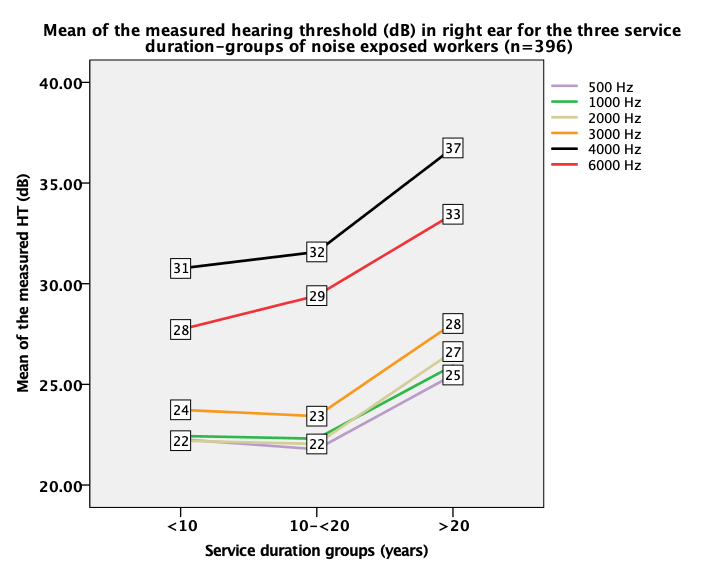 | | **Mean of the measured HT** (dB) | 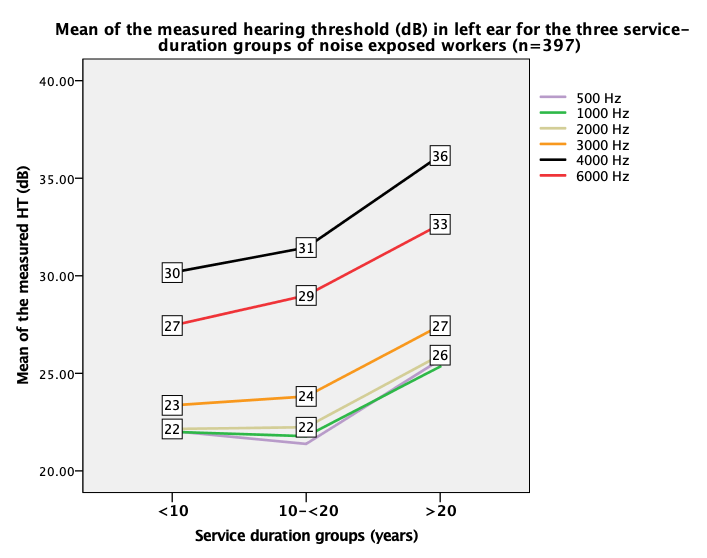 | | |
| --- | --- | --- | --- | --- | --- | --- |
|  | **A** | **Job duration groups** (years) |  | **B** | **Job duration groups** (years) | |
|  |  |  |  |  |  | |
| **Mean of the measured HT** (dB) | 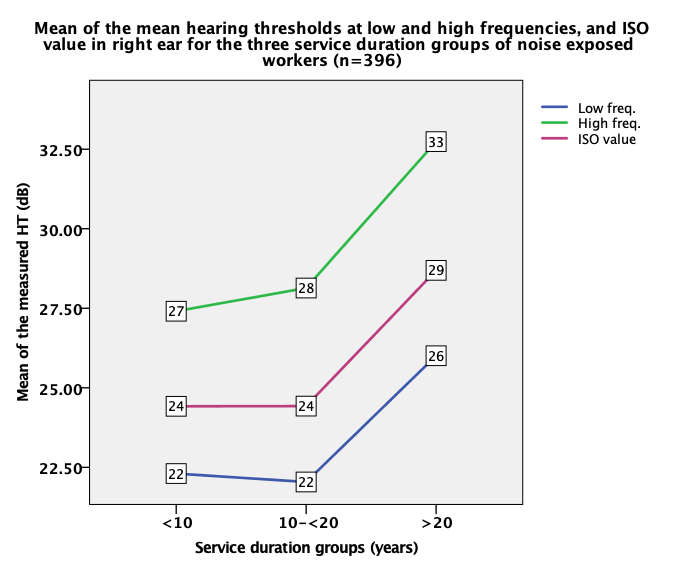 | | **Mean of the measured HT** (dB) | 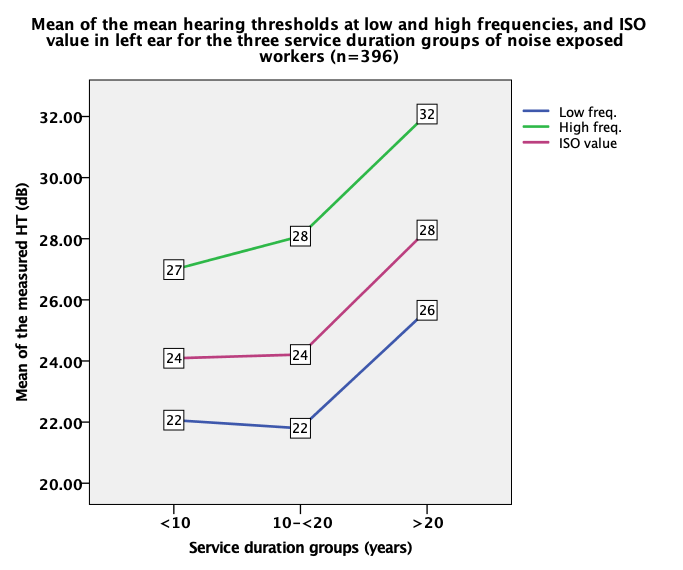 | | |
|  | **A** | **Job duration groups** (years) |  | **B** | **Job duration groups** (years) | |
|  |  | |  |  | | |
| **Figure S3: Mean of the measured hearing thresholds (dB) at specified audiometry frequencies (Hz) among noise-exposed workers (n=396) stratified by job duration, at two steel factories in Egypt, 2021**  Low frequencies: average of hearing thresholds at 0.5, 1, and 2 kHz  High frequencies: average of hearing thresholds at 3, 4, and 6 kHz  ISO value: average of hearing thresholds at 0.5, 1, 2, and 4 kHz  *Abbreviations: HT* hearing threshold; *dB* Decibel; *Hz* Hertz  A: right ear; B: left ear | | | | | |  |

| **Table S1: Multiple linear regression of predictors of hearing threshold at tested frequencies among noise-exposed workers (n=396) at two steel factories in Egypt, 2021** | | | | | | | | | | | | | | | | | | |
| --- | --- | --- | --- | --- | --- | --- | --- | --- | --- | --- | --- | --- | --- | --- | --- | --- | --- | --- |
| 1. Right Ear | | | | | | | | | | | | | | | | | | |
|  | Audiometry frequency (kHz) | | | | | | | | | | | | | | | | | |
|  | 0.5 kHz | | | 1 kHz | | | 2 kHz | | | 3 kHz | | | 4 kHz | | | 6 kHz | | |
|  | B^^^ | ß | 95%CI | B^^^ | ß | 95%CI | B^^^ | ß | 95%CI | B^ | ß | 95%CI | B^^^ | ß | 95%CI | B^^^ | ß | 95%CI |
| Age | 0.18 | 0.25 | (0.08, 0.29)^***^ | 0.10 | 0.15 | (0.01, 0.20)^*^ | 0.19 | 0.25 | (0.08, 0.29)^***^ | 0.16 | 0.19 | (0.04, 0.29)^**^ | 0.33 | 0.17 | (0.05, 0.61)^*^ | 0.37 | 0.23 | (0.14, 0.60)^**^ |
| Job duration | -0.30 | -0.03 | (-0.14, 0.08) | 0.06 | 0.08 | (-0.04, 0.17) | 0.04 | 0.06 | (-0.06, 0.16) | 0.04 | 0.05 | (-0.08, 0.18) | -0.002 | -0.001 | (-0.30, 0.30) | -0.04 | -0.02 | (-0.29, 0.20) |
| Tinnitus | 3.70 | 0.21 | (2.00, 5.40)^***^ | 0.83 | 0.17 | (1.3, 4.6)^***^ | 3.13 | 0.17 | (1.44, 4.83)^***^ | 4.37 | 0.21 | (2.39, 6.36)^***^ | 5.29 | 0.11 | (0.73, 9.85)^*^ | 5.16 | 0.13 | (1.4, 8.9)^**^ |
|  | R=0.30; R^2^=0.09; p<0.001 | | | R=0.28; R^2^=0.080; p<0.001 | | | R=0.34; R^2^=0.121; p<0.001 | | | R=0.31; R^2^=0.098; p<0.001 | | | R=0.20; R^2^=0.042; p=0.001 | | | R=0.24; R^2^=0.061; p<0.001 | | |

| 1. Left Ear | | | | | | | | | | | | | | | | | | |
| --- | --- | --- | --- | --- | --- | --- | --- | --- | --- | --- | --- | --- | --- | --- | --- | --- | --- | --- |
|  | Audiometry frequency (kHz) | | | | | | | | | | | | | | | | | |
|  | 0.5 kHz | | | 1 kHz | | | 2 kHz | | | 3 kHz | | | 4 kHz | | | 6 kHz | | |
|  | B^^^ | ß | 95%CI | B^^^ | ß | 95%CI | B^^^ | ß | 95%CI | B^^^ | ß | 95%CI | B^^^ | ß | 95%CI | B^^^ | ß | 95%CI |
| Age | 0.07 | 0.09 | (-0.03, 0.18) | 0.02 | 0.03 | (-0.07, 0.12) | 0.19 | 0.25 | (0.08, 0.30)^***^ | 0.20 | 0.23 | (0.08, 0.33)^**^ | 0.40 | 0.22 | (0.14, 0.67)^**^ | 0.36 | 0.24 | (0.15, 0.58)^**^ |
| Job duration | 0.08 | 0.10 | (-0.03, 0.20) | 0.11 | 0.15 | (0.01, 0.22)^*^ | 0.01 | 0.01 | (-0.10, 0.13) | 0.01 | 0.01 | (-0.12, 0.14) | -0.06 | -0.03 | (-0.35, 0.22) | -0.08 | -0.05 | (-0.31, 0.15) |
| Tinnitus | 4.15 | 0.23 | (2.40, 5.89)^***^ | 2.91 | 0.17 | (1.29, 4.53)^***^ | 4.12 | 0.22 | (2.38, 5.86)^***^ | 5.04 | 0.23 | (3.04, 7.04)^***^ | 5.36 | 0.12 | (1.07, 9.66)^*^ | 5.63 | 0.15 | (2.13, 9.1)^**^ |
|  | R=0.30; R^2^=0.091; p<0.001 | | | R=0.26; R^2^=0.069; p<0.001 | | | R=0.34; R^2^=0.118; p<0.001 | | | R=0.33; R^2^=0.114; p<0.001 | | | R=0.22; R^2^=0.052; p<0.001 | | | R=0.25; R^2^=0.065; p<0.001 | | |
| *Abbreviations: CI* confidence interval; *dB* Decibel; *Hz* Hertz  ^^^ adjusted for all variables in the above table (age and job duration as continuous variables, and tinnitus as dichotomous variable).  ^*^ p ≤ 0.05; ^**^ p < 0.01; ^***^ p < 0.001 | | | | | | | | | | | | | | | | | | |
